# The right to water in the slums of Mumbai, India

**DOI:** 10.2471/BLT.15.155473

**Published:** 2015-10-06

**Authors:** Ramnath Subbaraman, Sharmila L Murthy

**Affiliations:** a Division of Infectious Diseases, Brigham and Women’s Hospital and Harvard Medical School, 75 Francis St., PBB-A4, Boston, MA 02115, United States of America (USA).; b Suffolk University Law School, Boston, USA.

Attaining universal and equitable access to safe and affordable drinking water for all by 2030 (sustainable development goal 6)^1^ will be a major challenge, particularly in urban slum communities. In 2012, over 860 million people – about a third of the urban population of developing countries – were living in slums.^2^ The primary barriers to accessing water in slums are not solely monetary or technical but also legal, institutional, and political.^3^^,^^4^

In India, some slums are notified, or recognized, by the government. In some cities, notified slums are entitled to receive security of land tenure, which means that the people who live in them cannot be arbitrarily evicted. In other words, inhabitants have a form of property rights to the land even though they do not own it. People living in notified slums are also usually entitled to access city services, including connections to the water supply. In 2012, 59% of slum settlements in India were non-notified.^5^ People living in non-notified settlements suffer from poorer access to piped water, latrines, electricity and public transportation when compared to notified slums; they also receive considerably less assistance from the government’s slum improvement schemes.^5^

Similar policies linking water entitlements to land tenure compromise water access for people living in slums in cities in other low- and middle-income countries, including Bangladesh, Kenya and Nigeria.^6^^,^^7^ Even when sound public health and economic reasons exist for providing slums with access to municipal water supplies, lack of property rights can impede provision of this vital service. A recent court ruling from Mumbai, India, illustrates the nature of these legal and political barriers to water access and the potential of human rights law to overcome them.^4^

By some estimates, Mumbai has the largest slum population of any city in the world, with more than half of its 12 million people living in informal settlements.^5^ Nearly half of Mumbai’s slums are non-notified.^5^ The divide between notified and non-notified slums in Mumbai is tied to “cut-off” dates.^3^ Slum households who can prove that they have been living in a slum located on state or municipal land prior to a specified cut-off date can obtain notified status. This policy arose in response to democratic pressure from slum dwellers, who form a large proportion of Mumbai’s electorate. Currently, all slum households who settled on state or city government-owned land in Mumbai prior to the year 2000 can obtain notified status, while households who settled after the year 2000 remain non-notified. In addition, slums located on land in Mumbai owned by the central government do not benefit from this policy and remain non-notified despite having been established – in some locations – several decades ago.

Mumbai has a chlorinated central water supply managed by the government. People living in non-notified slums have historically been unable to legally connect to this system, forcing many of them to illegally tap into city water pipes out of desperation – a survival strategy that can compromise the safety of the water supply through cross-contamination.^5^

The consequences of exclusion from the water supply are illustrated by data from Kaula Bandar, a non-notified slum in Mumbai.^8^^–^^10^ This community was established more than 50 years ago but remains non-notified because it is on central government land. Since residents of Kaula Bandar are excluded from the formal water supply, they are forced to buy water from street vendors. In 2012, the median price paid for water by residents in the winter season was 135 Indian rupees (2.07 United States dollars) per 1000 litres of water.^8^ This was more than 40 times the standard municipal water charge paid by residents of notified slums and more than 30 times the charge paid by other city residents in 2014.^8^ Many residents of Kaula Bandar access less than 20 litres of water per person per day,^8^ which is below the minimum consumption level recommended by the World Health Organization for ensuring basic hygiene, particularly for women and children.^11^ Especially in the summer, drinking water is often contaminated with *Escherichia coli*, an indicator of faecal contamination.^9^

Lack of access to clean water causes diarrhoeal illness in children; in turn, recurrent diarrhoeal illness is associated with increased child mortality and poor nutritional status.^11^ Based on a comparison of data from a 2010 survey of 811 children in Kaula Bandar with India’s National Family Health Survey, the infant mortality rate in Kaula Bandar is more than twice that of other, mostly notified Mumbai slums and 30% higher than that of Mumbai’s formally housed population.^10^ In Kaula Bandar, about 46% of children younger than 5 years are moderately or severely underweight as compared to 36% in notified slums and 26% in Mumbai’s formally housed population.^10^

In December 2014, the Bombay High Court ordered the city government to extend access to Mumbai’s water supply to residents living in non-notified slums.^4^ This ruling was the result of years of public interest litigation by the group Pani Haq Samiti and is an important step forward for improving the health of two to three million residents in non-notified slums in Mumbai. Two aspects of the judgment may be particularly relevant for the public health community, as these arguments may inform efforts to advance water access in other cities in low- and middle-income countries. First, the court order uses a human rights-based framework, holding that the right to water is central to the right to life guaranteed by the Constitution of India. The order also cites international human rights law, in particular the United Nations International Covenant on Economic, Social, and Cultural Rights, which is the key basis of the human right to safe drinking water and sanitation under international law.^4^ Second, the court finds that water access should not be tied to the property rights of a slum, thereby disentangling security of tenure from the right to water.^4^ These two arguments allow the court to cut through what were previously considered intractable legal barriers to water access in non-notified slums.

Since the ruling, the city government has developed a new policy for supplying water to non-notified slum residents.^12^ However, the high court ruling and the new policy still have shortcomings. First, slums such as Kaula Bandar may continue to be excluded from the water supply because the Bombay High Court does not have jurisdiction over central government land, where some non-notified slums are located.^12^ Second, even while extending water to non-notified slums, the court has sanctioned a two-tiered system of access. The court states that in non-notified slums water need not be provided via individual home connections or at the same price as elsewhere in the city.^4^ Finally, the court does not address the underlying problem of security of tenure and emphasizes that the government remains obliged to eventually remove non-notified slums erected after the year 2000, in accordance with existing law.^4^ The court is therefore stating that until these settlements are removed, resettled, or rehabilitated, which could take years, if not decades, inhabitants should be provided with access to the city water supply.

Despite these limitations, the Bombay High Court decision underscores the fact that legal, institutional and political barriers are often greater obstacles to expanding water access than monetary or technical challenges, especially for poor urban communities.

Human rights-based frameworks that emphasize a universal right to water may play a valuable role in overcoming barriers for access to water and thereby help to promote health for marginalized urban populations. This is an important lesson as the global community endorses the goal of achieving universal and equitable access to water for all.^1^
